# Neural Mass Modeling in the Cortical Motor Area and the Mechanism of Alpha Rhythm Changes

**DOI:** 10.3390/s25010056

**Published:** 2024-12-25

**Authors:** Yuanyuan Zhang, Zhaoying Li, Hang Xu, Ziang Song, Ping Xie, Penghu Wei, Guoguang Zhao

**Affiliations:** 1 Department of Neurosurgery, Xuanwu Hospital, Capital Medical University, Beijing 100032, China; zyyflp12@163.com (Y.Z.); lizhaoying@mail.ccmu.edu.cn (Z.L.); xuhang@mail.ccmu.edu.cn (H.X.); songziang@mail.ccmu.edu.cn (Z.S.); 2 Key Laboratory of Measurement Technology and Instrumentation of Hebei Province, Institute of Electric Engineering, Yanshan University, Qinhuangdao 066004, China; pingx@ysu.edu.cn; 3 Key Laboratory of Intelligent Rehabilitation and Neuromodulation of Hebei Province, Institute of Electric Engineering, Yanshan University, Qinhuangdao 066004, China

**Keywords:** static grip force, cortical motor area, neural mass model, alpha rhythm

## Abstract

Investigating the physiological mechanisms in the motor cortex during rehabilitation exercises is crucial for assessing stroke patients’ progress. This study developed a single-channel Jansen neural mass model to explore the relationship between model parameters and motor cortex mechanisms. Firstly, EEG signals were recorded from 11 healthy participants under 20%, 40%, and 60% maximum voluntary contraction, and alpha rhythm power spectral density characteristics were extracted using the Welch power spectrum method. Furthermore, a single-channel neural mass model was constructed to analyze the impact of parameter variations on the average power of simulated signals. Finally, model parameters were adjusted to achieve feature fitting between the simulated signals and the average power of the alpha rhythm. Results showed that alpha rhythm average power in the contralateral cortical regions increased with higher grip force levels. Similarly, the power of the simulated signals also increased with specific parameter (*J*, *Ge*, and *Gi*) increases, closely approximating the measured EEG signal changes. The findings suggest that increasing grip force activates more motor neurons in the motor cortex and raises their firing rate. Neural mass modeling provides a computational neuroscience approach to understanding the dynamic changes in alpha rhythms in the motor cortex under different grip force levels.

## 1. Introduction

The motor cortex plays a pivotal role in controlling muscle movement by modulating the rhythmic oscillatory activity of motor neurons [1,2]. The cortical–muscular motor system adjusts hand force output by regulating the intensity, frequency bands, and direction of neuronal synchronous oscillations [3,4]. Consequently, different hand force outputs correspond to specific oscillatory activities in the motor cortex. Investigating the mechanisms underlying rhythmic changes in the motor cortex during varying handgrip forces is fundamental to understanding neuromuscular regulation and advancing clinical rehabilitation assessments.

Brown’s exploration of the fractal dimension (FD) of the motor cortex during varying grip levels revealed that the FD of electroencephalography (EEG) signals increased linearly with grip force during movement and gripping but showed no correlation during the preparation phase. This finding indicates that greater hand force intensifies the linear recruitment of motor neurons and their firing rate [5]. Similarly, Hao analyzed EEG characteristics in the time and frequency domains, finding that energy in the beta and gamma bands at 50% and 75% maximum voluntary contraction (MVC) exceeded that at 25% MVC. Their study emphasizes how handgrip levels modulate brain activity within specific frequency bands and cortical regions [6]. Fetz et al. also highlighted the relationship between distinct hand forces and corresponding oscillatory activities in the motor system [7]. Additionally, studies have shown that the power spectrum of the alpha band in EEG signals strongly correlates with grip strength activities [8,9]. Primarily, the alpha-band component reflects the level of attentional demand [10]. Ortiz et al. demonstrated that during grasping tasks with unexpected changes in object weight, alpha activity in the central region increased, while the frontoparietal network remained unchanged. This suggests that alpha oscillations may act as a biomarker for motor error correction [11]. Hou et al. further supported these findings by examining dynamic grip force regulation, finding that increased force demand significantly enhanced neural responses in the central cortex’s alpha and beta bands, while reducing the stability of muscle motor unit output [12]. Although these studies establish the relationship of hand force on fast cortical oscillations, the effects on slower oscillations, particularly the alpha rhythm, remain underexplored.

In parallel, computational neural models have become powerful tools for studying cortical neural oscillations, cortical organization, and neural information processing [13,14]. Neural mass models, in particular, encapsulate the collective behavior of neuron groups by using lumped state variables to represent the average dynamics of the neural network [15,16]. Jansen and Rit pioneered the development of single-channel and coupled dual-channel neural mass models to investigate brain rhythms, such as visually evoked potentials [17,18]. Building on this foundation, Wendling utilized genetic algorithms to achieve feature fitting between measured and simulated signals in terms of power spectral characteristics, thereby uncovering the physiological mechanisms underlying epileptic seizures [19]. Expanding on Wendling’s work, Ma et al. introduced an enhanced multi-channel neural mass model to explore seizure mechanisms, localize the epileptogenic zone, and control seizures [20]. These advancements highlight the ability of neural mass models to generate simulated signals that mirror measured EEG characteristics, offering a computational neuroscience framework for exploring cortical neural oscillations [21,22].

Building on the findings of previous studies, this work aims to establish a connection between the parameters of a single-channel Jansen neural mass model and the physiological mechanisms of the motor cortex. By fine-tuning the model parameters to achieve feature fitting between simulated and measured alpha rhythm average power signals, this study explores the intrinsic relationship between neural mass model parameters and the motor cortex physiology. These findings provide a theoretical foundation for understanding the dynamics of alpha rhythms in the motor cortex and their role in motor control during static grip force tasks.

## 2. Materials and Methods

### 2.1. Experimental Subjects and Paradigms

The EEG signal acquisition experiment involved 11 healthy participants, including 3 females and 8 males, with a mean age of 24.6 years (±3.0). All participants were right-handed and ensured adequate sleep prior to the experiment to minimize the potential impact of brain fatigue on the outcomes. The experiment was conducted in a laboratory environment specifically designed to shield against complex electromagnetic interference, ensuring EEG signals integrity. The experimental procedure was approved by the ethical review boards of Yanshan University and Xuanwu Hospital, Capital Medical University.

**Figure 1:**
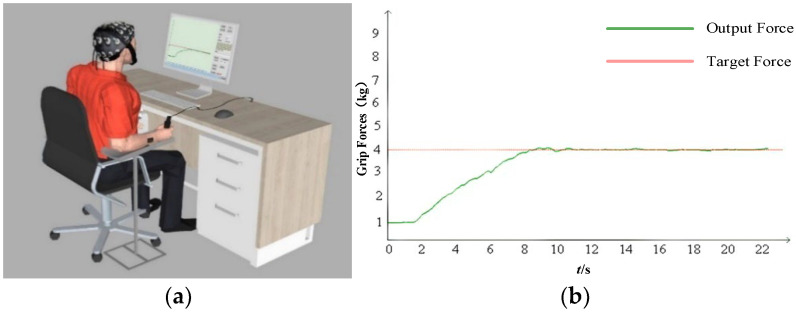
Synchronous acquisition of the electroencephalography (EEG) and maximum voluntary contraction (MVC) signals. (**a**) Recording of EEG data; (**b**) target force and actual output force display interface.

During the experiment, participants sat upright on the experimental platform and used their right hand to grasp a pressure sensor to measure grip strength, as shown in Figure 1a. A screen positioned approximately 80 cm from the participants displayed both the target force curve alongside and the output force curve generated by the pressure sensor. Participants were required to adjust their grip strength in real time to track the target grip force displayed on the screen, as depicted in Figure 1b.

**Figure 2:**
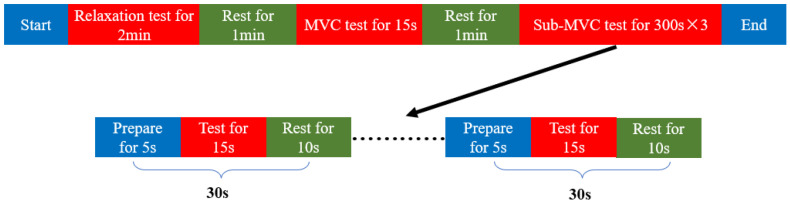
Experimental paradigm flow chart.

The specific experimental procedure is illustrated in Figure 2. The process began with the collection of EEG signals from healthy participants in a relaxed state for 2 min, followed by a 1 min rest period. Subsequently, the MVC force of the right hand was measured for each of the 11 participants. Each subject performed the MVC test for 5 s to ensure accuracy, repeating the task thrice. The MVC for each participant was determined by calculating the average of these three measurements.

EEG signals were then recorded at 20%, 40%, and 60% of the MVC for each participant. During the recording sessions, participants prepared for 5 s, maintained a stable grip strength for 15 s, and rested for 10 s. This sequence was repeated 10 times for each grip strength level. To prevent fatigue and ensure consistent performance, a rest period of 1–2 min was provided between transitions to different grip strength levels, adjusted to accommodate individual needs.

### 2.2. Data Recording and Preprocessing

The experiment employed the eego™sports system (ANT Neuro, Hengelo, The Netherlands) to collect 64-channel EEG signals, with electrodes positioned according to the 10–20 lead standard (Synamp2, Compumedics Inc., Charlotte, NC, USA). Participants were instructed to wash their hair before the experiment, don EEG caps, and apply conductive paste to the electrodes to minimize channel impedance and enhance the signal-to-noise ratio of the EEG recordings. EEG signals were recorded from 59 scalp locations, with the FCz electrode used as the reference.

Data analysis was performed offline using the MATLAB environment. During data acquisition, the EEG signal amplifier amplified the signals by a factor of 1000. The signals were filtered using a bandpass filter within a 0.5–200 Hz frequency range. EEG signals were sampled at a frequency of 1000 Hz, while pressure signals were sampled at 50 Hz. Consistent with the principle of contralateral limb control by the brain, the C3 channel was selected to represent the left cortical motor area responsible for right hand static grip strength output. Electromyographic (EMG) signals, particularly from muscles near the scalp such as those in the face or neck, can interfere with EEG signals when muscles contract. To avoid artifacts related to the initiation of force, data from the 10–20 s interval were used for analysis. Data were segmented into 300 s epochs, though no epoch was entirely artifact-free. Before artifact rejection, the data were further divided into 5000 ms nonoverlapping segments, yielding 90 segments per participant. Segments with large amplitude artifacts were discarded.

Due to the weak and interference-prone nature of EEG signals, a combined filter was implemented to remove artifacts from the raw EEG recordings, such as muscle electrical activity (EMG), electrooculogram (EOG), and 50 Hz power line noise. First, a high-pass filter was initially applied to remove baseline drift. Then, adaptive filtering was used to eliminate 50 Hz power frequency noise [23]. To address artifacts from electrooculography (EOG) and electromyography (EMG), Independent Component Analysis (ICA) based on Informax was performed [24]. Finally, Canonical Correlation Analysis (CCA) was employed to further reduce interference from EMG signals within the EEG data [25].

### 2.3. Power Spectrum Analysis of EEG Signals

The primary motor cortex is significantly activated during skilled movements of the contralateral limbs. Accordingly, EEG signals from the C3 channel, which corresponds to the primary motor area, were selected for analysis. Given the non-stationary and random nature of EEG signals, the smoothed periodogram method was employed to estimate the power spectrum and extract effective signal features in the frequency domain [26,27].

To begin, the EEG data were segmented using a non-rectangular window with overlapping segments. The N data points were divided into K segments, each containing M data points. Each segment was multiplied by a window function W(n), and the power spectrum for each segment was calculated. These segment power spectra were subsequently summed and averaged to produce the final power spectrum estimate. The power spectral density was computed using the following equation:

(1) $$S(\omega )=\frac{1}{K}\sum_{i=1}^{K}\frac{1}{MU}{\left|\sum_{n=0}^{M-1}X^{i}(n)W(n)e^{-j\omega n}\right|}^{2}$$

(2) $$U=\frac{1}{K}\sum_{n=0}^{M-1}W^{2}(n)$$

where K represents the number of data segments, M represents the length of each segment, $W\left(n\right)$ represents the window function, and U represents the average energy of the window.

Furthermore, the power spectral density S(ω) was integrated to calculate the average power *P* of EEG signals within each frequency band, as shown in Equation (3):

(3) $$P=\int_{\omega_{l}}^{\omega_{h}}S(\omega )d\omega$$

where $\omega_{h}$ and ${\;\omega }_{l}$ represent the lower and upper bounds of each frequency band, respectively.

### 2.4. Single-Channel Neural Mass Modeling and Analysis

#### 2.4.1. Single-Channel Neural Mass Model

**Figure 3:**
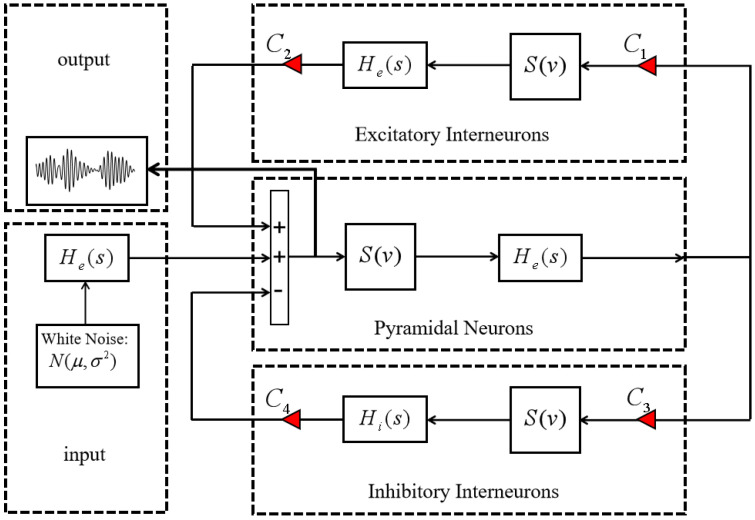
Composition of the single-channel neural mass model.

The basic single-channel neural mass model consists of three primary subpopulations: the pyramidal cell population, the excitatory interneuron population, and the inhibitory interneuron population [8], as illustrated in Figure 3. A static nonlinear function S(v) is employed to convert the average presynaptic membrane potential into the average spike density, representing the mean firing rate. This mean firing rate then serves as the input for the linear transformation function, as described in Equation (4):

(4) $$S(v)=\frac{2e_{0}}{1+e^{r(v_{0}-v)}}$$

The dynamic linear transformation functions $h_{e}(t)$ and $h_{i}(t)$ convert the average spike density into the average postsynaptic membrane potential. The unit step responses are described in Equations (5) and (6):

(5) $$h_{e}(t)=u(t)G_{e}\omega_{e}te^{-t\omega_{e}}$$

(6) $$h_{i}(t)=u(t)G_{i}\omega_{i}te^{-t\omega_{i}}$$

The transfer functions of the dynamic linear transformation functions $h_{e}(t)$ and $h_{i}(t)$, in combination with the static nonlinear function S(v), form three subpopulations: the pyramidal cell population, the excitatory interneuron population, and the inhibitory interneuron population. Gaussian white noise, processed through the excitatory dynamic linear transformation function $h_{e}(t)$, represents input signals from other regions. The parameters *C*_1_, *C*_2_, *C*_3_, and *C*_4_ represent the average number of synaptic connections between subpopulations within the neural mass model and are proportional to the parameter *J*, as shown in Table 1.

The excitatory and inhibitory interneuron populations receive excitatory feedback from the pyramidal cell population, while the pyramidal cell population receives both excitatory and inhibitory feedback from the excitatory and inhibitory interneuron populations, respectively [28]. Reciprocal interactions between the excitatory and inhibitory interneuron populations generate neuronal oscillations within the neural mass model. The presynaptic average membrane potential of the pyramidal cell population serves as the output signal of the neural mass model, representing the integrated expression of cortical neuronal oscillatory activity [29].

#### 2.4.2. Model Simulation and Parameter Settings

The neural mass model uses lumped parameters to represent the average behavior of the entire cell population within the neural network. The model can generate brain-like signals exhibiting different rhythms by adjusting these parameters. In MATLAB/Simulink, the model structure was simulated using the fourth-order Runge–Kutta method with a sampling frequency of 1000 Hz. Gaussian white noise (mean μ = 220; standard deviation σ = 22) was applied as the external input signal, and all model parameters were initialized to the typical values provided in Table 1. Adjustments to the parameters *G_e_*, *G_i_*, $\omega_{e}^{-1}$, and $\omega_{i}^{-1}$ regulated the dynamic balance between the excitatory and inhibitory neuronal populations, enabling the model to generate simulated EEG signals with alpha-like rhythms.

The single-channel neural mass model exhibits distinct types of dynamic behaviors depending on the values of the average synaptic connection number *J*, excitatory average synaptic gain *G_e_*, and inhibitory average synaptic gain *G_i_*. With all other model parameters set to the typical values (as detailed in Table 1), the effects of J, *G_e_*, and *G_e_* on the power spectral density of the model’s output signals were analyzed. Additionally, this study investigated how these parameters influence the average power of the alpha rhythm in the model’s output signals. Specifically, *J* was set with a baseline of 135 and adjusted in intervals of 0.1, *G_e_* was set with a baseline of 3.25 and adjusted in intervals of 0.001, and *G_i_* was set with a baseline of 22 and adjusted in intervals of 0.01.

## 3. Experimental Results

The primary motor cortex is significantly activated during skilled movement of the contralateral limbs [30,31]. EEG signals from the left motor area were selected for analysis. Initially, a segment of EEG data was extracted from the 5–15 s interval, during which the static grip force output remained relatively stable. Each data segment length contained 10,000 points. Following the method described in Section 2.2, EEG signals from 11 healthy participants were preprocessed under grip forces corresponding to 20%, 40%, and 60% MVC. Subsequently, the alpha rhythm (8–12 Hz) of the contralateral cortical regions’ signal was extracted using bandpass filtering.

### 3.1. Power Spectral Analysis of Alpha Rhythm Under Different Grip Forces

**Figure 4:**
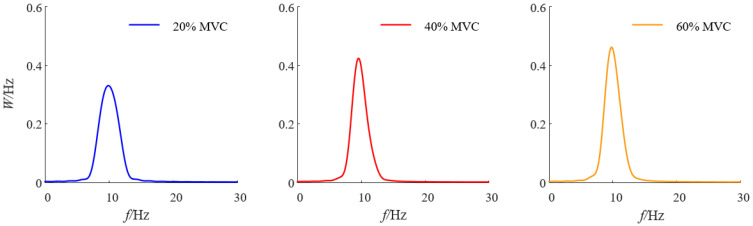
Power spectrum density analysis of alpha-band signals under three grip strengths.

Using the smoothed periodogram method described in Section 2.3, the alpha rhythm power spectral density was analyzed for three constant grip forces across the 11 participants. The power spectral density results for subject S1 under different static grip forces are shown in Figure 4. The analysis revealed that as grip force increased, the peak of the alpha rhythm power spectral density in the contralateral cortical regions of participant S1 also increased.

**Figure 5:**
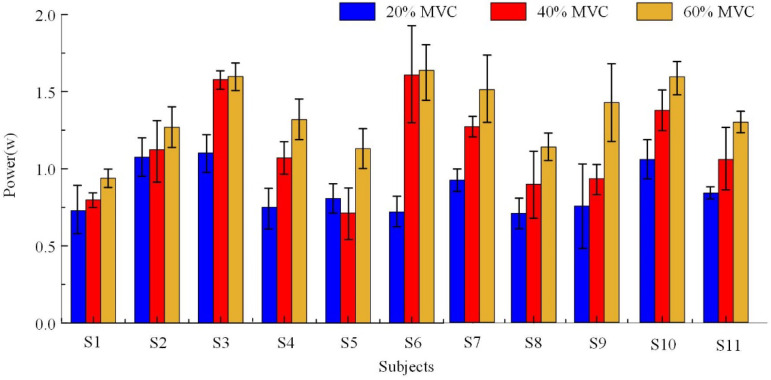
Power spectrum analysis of alpha bands under different grip strengths in 11 participants.

To provide a clearer representation of energy changes in the alpha rhythm under different constant grip forces, the power spectral density of the alpha rhythm for all 11 participants was integrated to calculate the average power of the alpha rhythm in the EEG signal. The results, shown in Figure 5, indicate that the average power of the alpha rhythm signal increased with grip force, despite individual variability. Notably, the average power of the alpha rhythm at 60% MVC was significantly higher than at 20% MVC across all 11 participants.

**Figure 6:**
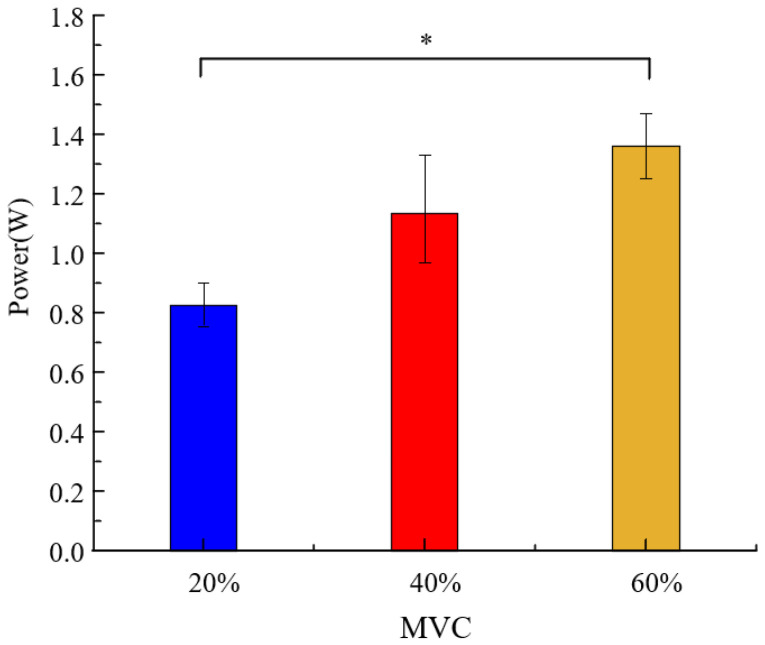
Power spectrum analysis of alpha-band signals under different grip strengths. The blue bars represent the power spectrum under 20% MVC, the red bars represent the power spectrum under 40% MVC and the yellow bars represent the power spectrum under 60% MVC. “∗” denotes *p* < 0.05.

To quantitatively assess the differences in average power among the three constant grip forces across the 11 participants, a one-way analysis of variance (*p* < 0.05) was conducted using SPSS 20.0 statistical software. This analysis evaluated changes in alpha rhythm average power under different grip forces. The data for the 11 participants at the three grip levels followed a normal distribution, as shown in Figure 6. The results indicate that as grip force increased, the alpha rhythm power in the contralateral cortical regions also increased. Specifically, the average power at 60% MVC was significantly higher than at 20% MVC (*p* < 0.05). This finding demonstrates that the average power of the alpha rhythm in the motor cortex increases with static grip force, suggesting a significant correlation between alpha rhythm activity in the motor cortex and static grip force.

### 3.2. Results of Neural Mass Model Parameter Analysis

**Figure 7:**
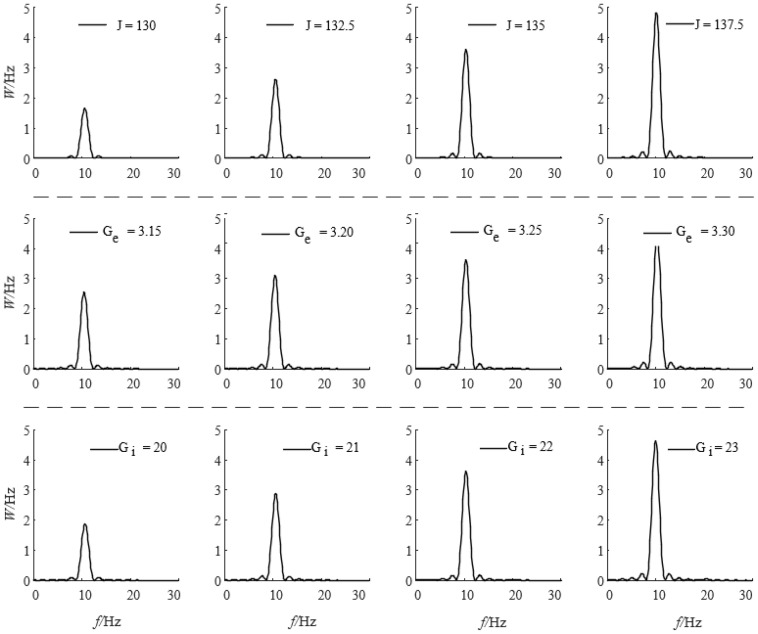
Analysis of the influence of neuron group model parameters on signal frequency spectrum density.

The characteristics of the output signals generated by the neural mass model are influenced by both external input signals and the values of the model parameters. This section examines the impact of the parameters *J*, *G_e_*, and *G_i_* on the power spectral density and average power characteristics of the model’s output signals. Using the typical parameters values for *J*, *G_e_*, and *G_i_*, which are listed in Table 1 as a baseline, each parameter was individually adjusted while maintaining the other model parameters at their baseline values. The effects of these adjustments on the power spectral density of the output signals were analyzed, as shown in Figure 7. The analysis indicates that increasing the value of *J* results in a higher peak in the power spectral density of the model’s output signals without causing a shift in the peak frequency. This finding suggests that *J*, the average number of synaptic connections between the pyramidal cell population and the excitatory or inhibitory interneuron populations, directly influences the power characteristics of the model’s output signals. Similarly, increasing the excitatory average synaptic gain *G_e_* and inhibitory average synaptic gain *G_i_* leads to a gradual increase in the peak of the power spectral density of the model’s output signals, again without shifting the peak frequency. These results demonstrate that the synaptic gains *G_e_* and *G_i_* of the model’s subpopulations significantly affect the power characteristics of the model’s output signals.

**Figure 8:**
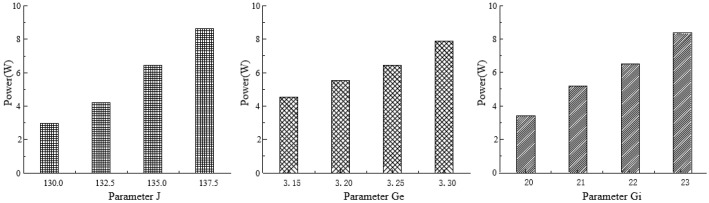
Analysis of the influence of neural mass model parameters on signal power spectrum. The different shading patterns in the figure represent the changes in power values corresponding to the variations in parameters *J*, *G_e_*, and *G_i_*.

To further analyze the impact of the model parameters on the average power of the output signals, the power spectral density of the model’s output signals was integrated. The results, as shown in Figure 8, indicate that the average power of the model’s output signals gradually increases with increases in the parameters J, $G_{e}$, and $G_{i}$.

### 3.3. Results of Feature Fitting Between EEG Signals and Simulated Model Signals

**Figure 9:**
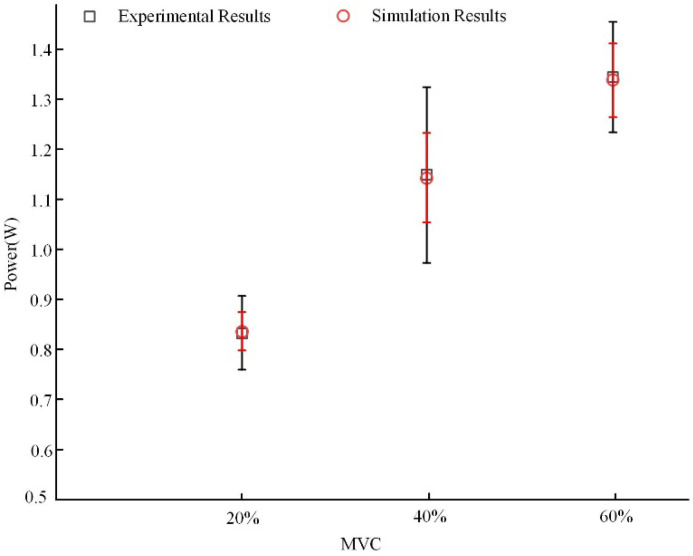
Comparative analysis of actual measurement results and simulation results.

**Figure 10:**
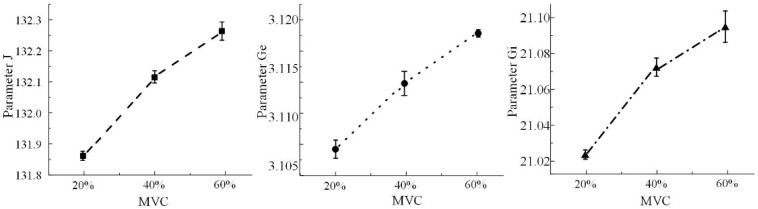
Analysis of model parameters of neuron groups for different grip strengths.

To achieve feature fitting between the measured EEG signals and the simulated signals from the neural mass model, the average alpha rhythm power of the contralateral cortical regions for 11 participants under three constant grip forces was calculated using the method described in Section 2.3. This average alpha rhythm power served as the characteristic feature of the measured signals. Simultaneously, the model parameters *J*, *G_e_*, and *G_i_* were adjusted to align the simulated signals with the alpha rhythm signals of each of the 11 participants in terms of average power characteristics. The results, shown in Figure 9, indicate that synchronously adjusting the neural mass model parameters *J*, *G_e_*, and *G_i_* enables feature fitting between the simulated and measured signals based on average power characteristics. Furthermore, the relationship between the model parameters *J*, *G_e_*, and *G_i_* and the constant grip force is illustrated in Figure 10. These results demonstrate that the parameters increase progressively with increasing grip force.

## 4. Discussion

Using the single-channel Jansen neural mass model, a connection between the model parameters and the physiological mechanisms of the motor cortex was established. By adjusting the model parameters, feature fitting between the simulated signals of the neural mass model and the measured EEG signals was achieved. This facilitated an exploration of the relationship between the neural mass model parameters and the physiological mechanisms of the brain’s motor cortex.

The findings of this study suggest that, as grip strength increases, the average power of alpha rhythms in the contralateral cortical regions progressively increases. Furthermore, changes in hand grip levels appear to modulate brain rhythmic activity in relevant cortical regions and specific frequency bands. Yao et al. have demonstrated that changes in hand grip levels can regulate brain rhythmic activity in corresponding cortical regions and specific frequency bands. EEG energy in related frequency bands tends to be greater during 50% MVC, 75% MVC, and fatigue states compared to 25% MVC [32]. Studies have also suggested that, in static force states, Granger causal connectivity from the cortex to the muscles tends to be significantly enhanced in the beta and high-alpha frequency bands [33,34]. This finding aligns with previous studies. Additionally, the rate of force variation has been suggested to have a greater impact on the response of the contralateral motor cortex in the alpha band. Specifically, as the frequency of target force variation increases, the average alpha-band event-related desynchronization also tends to increase [12]. Alpha-band activity is assumed to reflect attentional demands, being not only suppressed by sensory stimulation and movement but also modulated by attention, working memory, and mental tasks, with potential sensitivity to higher motor control functions [35,36,37]. In our study, the increase in grip strength appears to prompt the participants to accelerate necessary cognitive processes to adapt to changes in dynamic tasks, leading to the recruitment of more motor neurons and an increase in their firing rates, which consequently alters the power characteristics of the alpha rhythm in motor cortical EEG signals.

By fitting the alpha rhythm average power features of EEG signals and the simulated signals from the neural mass model under three different constant grip forces, the relationship between the neural mass model parameters and the physiological mechanisms of the motor cortex was explored. The fitted model parameters *J*, *G_e_*, and *G_i_* were found to increase synchronously with increasing grip force. In the neural mass model, the parameters *C*_1_, *C*_2_, *C*_3_, and *C*_4_ represent the average number of synaptic connections between subpopulations, which are proportional to the parameter *J*. The increase in the average number of synaptic connections in the model suggests an increase in the number of neurons in the pyramidal cell population and the excitatory and inhibitory interneuron populations [38], indicating that as grip force increases, the motor cortex recruits more motor neurons [39]. Postsynaptic potentials, including excitatory and inhibitory postsynaptic potentials [38], summate at their generation sites. EEG signals result from the total postsynaptic potentials generated by a large number of cortical neurons [6,40]. Previous studies have shown that in the dynamic linear transformation functions $h_{e}(t)$ and $h_{i}(t)$ of the neural mass model, changes in excitatory [41] and inhibitory average synaptic gains *J*, *G_e_*, and *G_i_* affect the peak of the average postsynaptic membrane potential [42]. The average synaptic gains *G_e_* and *G_i_* influence the firing rate of the postsynaptic membrane, thereby altering the power characteristics of the simulated output signals. This modulation enables feature fitting between the simulated and measured EEG signals, highlighting the strong connection between the alpha rhythm in the motor cortex and motor control. This understanding provides valuable insights into the pathological mechanisms underlying stroke and the synaptic remodeling processes in the cortex during rehabilitation. By elucidating the interplay between synaptic parameters and motor neuron recruitment, the model offers a framework for investigating motor control and its restoration in clinical contexts.

## 5. Conclusions

In this study, we have developed a single-channel Jansen neural mass model to investigate the relationship between model parameters and motor cortex mechanisms, particularly in relation to changes in alpha rhythm power during different grip force levels. The results suggest that increasing grip force leads to greater motor neuron recruitment in the motor cortex, which is reflected by an increase in the average power of the alpha rhythm in the contralateral cortical regions. Moreover, the neural mass model successfully approximated the changes observed in EEG signals by adjusting key model parameters (*J*, *G_e_*, and *G_i_*), suggesting that increasing grip force activates more motor neurons in the motor cortex and raises their firing rate. These findings provide valuable insights into the mechanisms underlying motor control tasks and the modulation of alpha rhythms in the motor cortex. And the model’s ability to capture the effects of grip force on neuronal oscillations lays the foundation for future investigations into synaptic remodeling in stroke patients’ motor control areas.

## Figures and Tables

**Table 1:** Neural mass model parameters.

Parameter	Physiological Significance	Alpha Rhythm
$G_{e}$	Excitatory Average Synaptic Gain (mV)	3.25
$G_{i}$	Inhibitory Average Synaptic Gain (mV)	22
$\omega_{e}^{-1}$	Excitatory Membrane Potential Average Time Constant (s)	0.0108
$\omega_{i}^{-1}$	Inhibitory Membrane Potential Average Time Constant (s)	0.02
J	Average Number of Synaptic Connections $C_{1}=J,C_{2}=0.8\times J,C_{3}=C_{4}=0.25\times J$	135
$e_{0},v_{0},r$	Static Nonlinear Function Parameters S(v)	$\begin{array}{l} e_{0}=2.5\;s^{-1},v_{0}=6\;mV\\ r=0.56\;mV^{-1} \end{array}$

## Data Availability

All data that support the findings of this study are available on request from the first author.
